# 

**Published:** 1844-10

**Authors:** 


### BOOKS RECEIVED FOR REVIEW.

1. Observations on the Epidemic Fever of 1843 in Scotland. By W. P. Alison, M.D.—Edinburgh, 1844. 8vo. pp. 80.

2. Thoughts on Physical Education, &c. By C. Caldwell, M.D. With a Preface by George Combe. Second Edition. — Edinburgh, 1844. Royal 8vo, pp. 36. 1s.

3. An Address delivered before the Dublin Obstetrical Society, December 1843. By W. F. Montgomery, M.D.—Dublin, 1844. 8vo. pp. 23.

4. On the best Modes of representing accurately by Statistical Returns, the Duration of Life, &c. By Edwin Chadwick, F.R.S.—London, 1844. (From the Statistical Journal.)

5. Catalogue of the Museum attached to the Class of Military Surgery in the University of Edinburgh.—Edin., 1844. 8vo, pp. 37.

6. Cases of Deformity of various kinds, successfully treated by Plastic Operations. By T. D. Mütter, M.D. — Philadelphia, 1844. 8vo, pp. 38.

7. A Report on the Operations for Fissures of the Palatine Vault. By T. D. Mütter, M.D.—Philadelphia, 1843. 8vo, pp. 28.

8. Cases of Deformity from Burns successfully treated by Plastic Operations. By T. D. Mütter, M.D.—Philadelphia, 1844. 8vo, pp. 24.

9. Zur Vermittelung der Extreme in der Heilkunde. Von Theodor Stürmer, M.D., &c.—Vols. 1, II, III.—Leipzig, 1837-9-43.

10. Actonian Prize Essay, Chemistry as Illustrative of the Wisdom and Benificence of God. By E. Fownes.—London, 1844. 8vo, pp. 184.

11. A Pratical Treatise on Midwifery. By M. Chailly, M.D. Translated from the French By G. S. Bedford, A.M. M.D.—New York, 1844. 8vo, pp. 526. With 216 Woodcuts.

12. The Practice of Medicine; a Treatise on Special Pathology and Therapeutics. By R. Dunglison, M.D., &c. Second Edition. In Two Volumes 8vo.—Philadelphia, 1844.

13. Defence of Hahnemann and his Doctrines, including an Exposure of Dr. A. Wood's Homœopathy Unmasked.—London, 1844. 8vo, pp. 92.

14. On the Decrease of Diseases effected by the progress of Civilization By C. F. H. Marx, M.D. and R. Willis, M.D.—London, 1844. 12mo, pp. 102.

15. Graefenberg; or a true Report of the Water Cure, with an Account of its Antiquity.—London, 1844. 8vo, pp. 322.

16. Narrative of a Voyage to Madeira, Teneriffe, &c. By W. R. Wilde, M.R.I.A. Second Edition enlarged.—Dublin, 1844. 8vo, pp. 648.

### INDEX TO VOL. XVIII

OF THE

### BRITISH AND FOREIGN MEDICAL REVIEW.

PAGE

Addison, Mr. on nutrition . . 91
Dr. on pneumonia . . 337
Alcohol, quantity drunk in different countries . . . 116
Alison, Dr. on fever . . . 179
his practice of medicine . 213
on the condition of the poor . . . 492
Almonds, oil of bitter, poisoning by . 560
Amaurosis, interesting cases of . 414
danger of depletion in . 415
Amussat, M. on artificial anus . . 452
Anatomy and histology, special . 223
Anatomy of expression, Sir C. Bell on 442
Anchylosis, Dr. Little on . . 353
false, M. Dancel on . ib.
Animal irritant poisons . . 555
Anus, artificial, various treatises on . 452
Armstrong, Dr. on climate . 221
Arsenic, on poisoning by . . 536
on tests for . . ib.
Articular cavities, pathology of . 82
Astragalus, on dislocations and extirpations of . . 124
on dislocations of . 565
Ague, effect of civilization on . 253
Barlow, Dr. obituary of . . 281
Barras, Dr. on gastralgia . . 208
Bell, Sir Charles, on expression . 442
Bethlehem hospital, statistics of . 371
Blood, changes of in inflammation . 255
its state in Bright's disease . 336
Blondlot, M. his treatise on digestion 529
Boeck and Danielsen on leprosy . 346
Bone, on the regeneration of . . 529
Books received for review . 284-570
Brain, on the fibres of . . 141
Dr. Foville's anatomy of . 423
and nerves, offices of . . 532
Brodie, his case of tracheotomy . 370
Bronchus, foreign body in . . ib.
Brunel, Mr. his case . . ib.
Burns, on deformity from . . 525
Calculus, bronchial, case of . 365
biliary, large . . 366
Calculi in St. George's hospital . ib.
Callisen, M. on artificial anus . 462
Caloric, Dr. Metcalfe on . . 563
Cancer of the uterus . . 30
stomach, treatise on . 208
rectum, M. Vidal on . 452
Cancrum oris, Dr. Hunt on . 367

PAGE

Cancrum oris, its medico-legal relations 543
Cannabis sativa, medicinal properties of 368
Carpenter, Dr. on white corpuscles . 569
Catalepsy, effect of civilization on . 250
Cataract, remarks on . . 416
operation for . . 217
Cavities, close, the pathology of . 79
Cecum, inflammation of . . 312
Cells, on the pathology of . . 339
Cellular cavities, pathology of . . 82
Chadwick, Mr. on the duration of life 197
Chimney-sweeper's cancer, obsolete . 253
Children, Dr. Rees on . . 236
Chemistry as bearing on theology . 531
Chevers, Dr. on chirurgical deaths . 333
Dr. on the coronary arteries 334
Children, health of, in cotton mills . 147
Chorea, effect of civilization on . 249
Choroiditis, Van der Kolk on . 48
Civilization, its effects on health . 237
Claessen, Dr. on diseases of the pancreas . . . 388
Clendinning, Dr. on Indian hemp . 368
Clergyman's sore throat . . 294
Climate, influence of on health . 221
Club-foot, treatise on . . 353
Colica pictonum, effect of civilization on . . 251
means of preventing 549
Congestions, effect of civilization on . 251
Consumption in Ireland as a cause of death. . 39
effect of civilization on 246
Convolutions of the brain, nature of . 432
Convulsions, puerperal, case of . 340
Copper, on poisoning by . . 549
Coronary arteries, diseases of . . 334
Coroner's inquests, remarks on . 43
Cormack, Dr. on fever . . 179
Corpus callosum, anatomy of . 430
Corpuscles of the blood, Mr. Addison on . . 92
white, on functions of . 569
Cotton-mills of Austria, account of . 147
Craigie, Dr. on fever . . 179
Croup, effect of civilization on . 251
Valleix and Dunglison on . 300
Dalrymple, Mr. on tumours . . 369
Dancel, M. on false anchylosis . 353
Danielsen and Boeck on leprosy . 346
Death, on the causes of, in England 197
sudden, Dr. Alison on . 213

PAGE

Death, sudden, modes of . . . 214
Deformity from burns, Dr. Mütter on 525
Delirium tremens, effect of civilization on . . . 250
Development of mammalia . 233
Diabetic blood, on sugar in . . 369
Digestion, M. Blondlot's treatise on 529
Diseases, how influenced by civilization 237
Dislocations of the astragalus, on . 124
Dancing mania, effect of civilization on 250
Donné, Dr. on the milk of nurses . 155
Dover's powder, poisoning by . 557
Drainage, reports on . . 493
Dumas and Boussingault on organic chemistry . . . 531
Dunglison, Dr. his practice of medicine 285
 treatise on physiology 530
Duval, M. on clubfoot . . 353
Dysentery, effect of civilization on . ib.
 M. Puchelt on . . 316
Dysmenorrhea, Dr. Rigby on . 522
Ear, on the pathology of . . 370
Elephantiasis græcorum . . 346
Encysted tumours, Mr. Dalrymple on 369
Enteritis, Puchelt on . . 311
Erectile tumour, Mr. Liston's case of 367
Erichsen, Mr. on congestive pneumonia . . . 365
Expression, anatomy and physiology of . . . 442
Eye, on internal inflammation of . 48
 treatise on diaeases of . 405
Factory system, effects of on health . 147
Farr, Mr. on vital statistics. . 197
Fatty degeneration of arteries . 366
Feculent discharge from umbilicus . 340
Feigned diseases, causes of . 119
Feigned or factitious diseases, treatises on . . . 117
Fever in Ireland as a cause of death . 38
 epidemic in Scotland . . 179
 effect of civilization on . 252
 report on cases of . . 337
Fibrin in inflammatory blood, source of 260
Finns, form of their skull . . 375
Foville, Dr. his anatomy of the brain 423
Fownes, Mr. his prize essay . . 531
Frank, Joseph, his Prax. Med. . 310
Ganglial cavities, pathology of . 83
Ganglionic nerves, microscopic anatomy of . . . 142
Gastralgia and gastritis, on . 208
Gavin, Dr. on feigned diseases . 117
Gilders' disease, effects of civilization on . . . 250
Glanders in man, case of . . 335
Glasgow, sanitary report of . 492
Glaucoma, on the cause of . . 48
Great Britain, public hygiene of . 492
Greenhill, Dr. his edition of Sydenham 527
Greenlanders, form of their skulls . 378
Griffiths, Dr. on the urine . 228
Grinders' asthma, Dr. Holland on . 302

PAGE

Guide to medical practice . . 285
Gulliver, Mr. on diseased arteries . 366
Guy's hospital reports . . 332
Harrison, Dr. on the nervous system 232
Hannover, Dr. on the nervous system 134
Headach, periodical . . 65
Healing process, Mr. Travers on . 69
 Mr. T. W. Jones on 255
 nature of . . 268
Hemorrhoids, treatment of . 34
Henderson, Dr. on fever . . 179
Hernia, report of cases of . . 336
 strangulated, Mr. Luke on . 367
Histology and anatomy . . 223
Hodgkin, Dr. on adventitious structure 369
Hoeven, Dr. Van der, on the negro race . . . 372
Holland, Dr. on diseases of the lungs 302
Horner, Dr. on anatomy . . 223
Hughes, Dr. on paracentesis thoracis 343
Hunt, Dr. on tic douloureux . . 56
Hunt, Dr. on cancrum oris . 367
Hydatids of the uterus . . 22
Hydrocele, encysted, Mr. Liston on]. 369
Hydrometra uteri . . . 21
Hydrophobia, effect of civilization on 250
Hygiene of Great Britain . . 492
Hyperinosis of the blood . . 257
Hypochondriacs, effect of civilization on . . . 249
Hypoglossus nerve, origin of . 136
Hysteria, effect of civilization on . 249
Hysterical affections of the voice . 367
Indian hemp, medical effects of . 365
Inflammation, Mr. Travers on . 69
 Dr. Williams on . 91
 nature of . . 103
 termination of . 268
 Mr. T. W. Jones on . 255
 resolution of . . 279
 effect of civilization on 251
Intemperance in Ireland, deaths from 41
Intermittent fever, physiology of . 235
Intestines, treatise on diseases of . 310
Invalid, essays by an . . 472
Ireland, on the causes of death in . 35
Jeaffreson, Mr. on diseases of the eye 405
Jones, Mr. T. W. on inflammation . 255
Jones, Dr. B. on diabetic blood . 369
 on calculi . . 366
Jugular vein, ulceration of . . 367
Jukes, Mr. on stricture of the rectum 452
Kafirs, form of their skulls . 386
King, Mr. on rupture of œsophagus . 335
Kidneys, experiments on . . 366
Kolk, Van der, on inflammation of the eye . . . . 48
Laplanders, form of their skulls . 376
Laryngitis, Valleix and Dunglison on 287
 acute, account of . . ib.
 chronic, account of . 291
 œdematous . . . 296
 stridulous . . . 298

40

PAGE
Laycock, Dr. on proleptics . . 165
Lead, on poisoning by . . 546
poisonous action of water on . ib.
Lepra, effect of civilization on . 254
Leprosy, Norwegian, account of . 346
Lever, Dr. on puerperal convulsions . 340
pelvic inflammation . 341
Life in the sick room . . 472
Luke, Mr. on strangulated hernia . 367
Lisfranc, M. his clinical surgery . 1
Liston, Mr. his case of erectile tumour 367
on encysted hydrocele . 369
Little, Dr. on anchylosis . . 353
Littré, M. on artificial anus . . 461
Longevity and regimen, Dr. Bell on 115
Lugol, M. on the cause of scrofula . 481
Louis on phthisis, translation of . 526
Lungs, diseases of, from dust, &c. . 302
Mackenzie, Dr. on fever . . 179
Malaria of Glasgow, effects of . 510
Martineau, Miss, her essays . . 472
Marx, Dr. on the effects of civilization 237
Medical science, essay on the nature of 235
Medicine, Dr. Alison's treatise on . 213
practical, treatise on . 285
Medico-chirurgical Transactions, vol. xxvi. . . . . . . 364
Medulla oblongata, on the functions of 134
anatomy of . 427
Memoirs of the Royal Academy of Medicine . . . . 152
Meningitis, epidemic . . . 160
Mercury, on poisoning by . . 542
Metcalfe, Dr. on caloric . . 513
Meyer, Dr. on nervous fibrils . 134
Miasmata of towns, ill effects of . 502
Milk of nurses, examination of . 155
Moles of the uterus . . . 18
Morbidity of mind, remarks on . 472
Mortality of Glasgow, report on . 492
Mortification, nature of . . 268
Narcotico-irritant poisons, report on 562
Negroes, form of their skulls . 384
Nerves, Foville on the origin of . 439
Nervus accessorius . . . 136
Nervous diseases, Dr. Rowe on . 229
system, on the structure of . 134
general physiology of 144
new theory of . 232
Neuralgia from anemia . . 63
from spinal irritation . ib.
uterine disease . 64
disease of the brain . 65
mechanical causes . ib.
Dr. Hunt on . . 56
description of . . 57
from dyspepsia . . 59
Neuralgia, from disordered liver . 62
effect of civilization on . 250
Nostalgia, effect of civilization on . 249
Norwegian leprosy . . . 346
Nurses, examination of the milk of . 155
Nutrition, on the process of . . 91

PAGE
Occupation, its relation to health . 92
Œdema glottidis . . 296
Œsophagus, digestive solution of . 335
Oldham, Dr. on polypus uteri . . 344
Ophthalmia, effect of civilization on . 251
Ophthalmitis interna . . 51
post-febrile, Dr. Mackenzie on . 192
Opium, recovery from a large dose of 557
Pancreas, treatise on diseases of . 388
Pancreas and stomach, diagnosis of diseases of . . . 398
Paracentesis thoracis, Dr. Hughes on 343
Paralysis, Dr. Webster's case of . 369
Pelvic tumours, Dr. Lever on . 333
inflammation, Dr. Lever on . 341
Pessaries, use of . . 27
Periodicity on, in health and disease . 165
Schwann and Quetelet on ib.
Peruvians, form of their skulls . . 380
Pettigrew, Mr. on medical superstitions 234
Phthisis at Martinique . . 158
Phosphorus, on poisoning by . . 536
Physiology, Dr. Dunglison's human . 530
Physometra of the uterus . . 19
Perry, Dr. his sanitary report . 492
Plague, effect of civilization on . 252
Pneumonia and its consequences . 337
congestive, Mr. Erichsen on 365
Poisoning, case of irritant . . 332
cases of, by Mr. Taylor . 342
antidotal treatment of . 553
Poisons, irritant, report on . 533
narcotic, report on . 557
Polypus of the uterus . . 5
vulva . . 17
vagina . . ib.
uteri, Dr. Oldham on . 344
Poor in Scotland, condition of . 492
Principles of medicine, Dr. Williams's 91
Process of nutrition, nature of . 101
Procter, Dr. on the sympathetic nerve 528
Proleptics, contributions to . 165
Provisions, price of, effect on health of 155
Prussic acid, new antidote for . 553
cases of poisoning by . 559
Puchelt Dr. on diseases of the intestine 310
Pulmonary emphysema a cause of death 162
arteries, ulceration of . 367
Quinine, its value in intermittents . 162
poisonous properties . 164
Raphania, effect of civilization on . 250
Rectum, cancer of, treatise on . 452
Rees, Dr. on the blood . . 336
Regimen and longevity, Dr. Bell on . 115
Registrar-general, his report . 197
Respiratory nerves, structure of . 136
Retzius, M. on the form of skulls . 372
Rickets, effect of civilization on . 247
on the skull . 371
Rigby, Dr. on dymenorrhea . 522
Robinson, Mr. on the renal vessels . 366
Rognetta, M. on the astragalus . 124
Rowe, Dr. on nervous diseases . 229

PAGE
Rufz, Dr. on phthisis at Martinique 158
Sanitary Commission, reports of . 492
Schweig, Dr. on periodicity . 165
Sciatica, Dr. Hunt on . . 566
Scrofula, Dr. Smith on . . 231
effect of civilization on . 247
M. Lugol on the causes of . 481
hereditary origin of . 482
pathological causes . . 489
external causes of . . 490
the result of bad ventilation. 500
Scurvy, effect of civilization on . 253
Self-consciousness, a modern disease. 479
Serous cavities, pathology of . 87
Shaw, Mr. on rickets . . 371
Skull, effect of rickets on . . ib.
on the national forms of . 372
Slavonians, form of their skulls . 375
Smallpox, effect of civilization on . 254
Smith, Dr. T. on scrofula . . 231
Soldiers, on the enlisting of . 117
Spermatozoa in hydrocele . . 369
Spinal cord, Foville's anatomy of . 425
Spine, on curvature of . . 225
Sponges, use of in displacement of uterus . . . . . . 26
Stafford, Mr. on curvature of spine . 225
Stilling on the medulla oblongata . 134
Stomach, on cancer of . . . 208
Stomach and pancreas, diagnosis of diseases of . . . 398
Strabismus, operation for, remarks on 414
Structures, adventitious, Dr. Hodgkin on . . . 369
Superstitions, medical, Mr. Pettigrew on . . . . . 234
Surgical operations, causes of death in 333
Sweating sickness, epidemic . 162
Swedes, form of their skulls . . 373
Sydenham Society, publications of . 526
Sydenham's works by Dr. Greenhill ib.
Sylvius, fissure of . . 435
Sympathetic nerve, treatise on . 528
Syphilis, effect of civilization on . 248
Swan, Mr. on the brain and nerves . 532
Tapeworm, Dr. Wawruch on . 319
diagnosis of . . 322
etiology of . . 323
prognosis of . . 326
treatment of . . 328
Taylor, Mr. on irritant poisons . 332
cases of poisoning by . 342
his report on toxicology 533
Teheran, epidemic at . . 369
Temperaments, M. Roger Collard on 153
Textor, M. on regeneration of bone . 529
Tic douloureux, Dr. Hunt on . . 56
Tice, Mr. his case of bronchial calculus . . . 365
Todd, Dr. his experiments . . 69

PAGE
Tongue, Dr. Williams on . 236
Toynbee, Mr. on the ear . . 370
Toxicology, report on the progress of 533
Tracheotomy, case of . . 370
Travers, Mr. on inflammation . 69
Tschudi, M. on the form of skulls . 372
Tumours, recto-vaginal . . 18
Turner, Mr. on the astragalus . 124
reply to his reclamation 565
Tympanitis of the uterus . . 19
Typhlitis and perityphlitis . . 312
Ulceration of the pulmonary artery . 367
Urine, Dr. Griffiths on the examination of . . . 228
Urine, albuminous, remarks on . 336
Uterus, diseases of, M. Lisfranc on . 1
fibrous tumours of . ib.
polypus of . . 5
dropsy of . . . 21
hydatids of . . 22
inversion of . . . 22
prolapsus of . . 23
retroversion of . . 24
anteversion of . . 25
obliquity of . . 26
anteflexion of . . ib.
morbid fixity of . . 27
affections of the cervix of . 28
ulcers of . . . . ib.
cancer of . . . . 30
amputation of neck of . 32
Vagina, explosion of gas from . 21
Vagus nerve, origin of . . . 137
functions of . . 139
Valleix, M. on pathology and therapeutics . . . . . 285
Velpeau, M. on close cavities . . 79
Ventilation, its relation to health . 498
means of improving . 501
Ventricle, fourth, anatomy of . . 137
Verdigris, on poisoning by . . 550
Vital phenomena, periodic, on . 166
Vital statistics of England . . 197
Vrolik, Dr. on development . 233
Walshe, Dr. his translation of Louis . 526
Water of towns, in relation to health 495
Watt, Dr. his Glasgow bills of mortality . . . 492
Wawruch, Dr. on tapeworm . 319
Webster, Dr. his case of paralysis . 364
his statistics of Bethlem 271
Wilde, Mr. on death in Ireland . 35
Williams, Dr. his principles of medicine . . 91
on medical science . 235
on cells . . 339
Women, diseases of, Dr. Rowe on . 229
Wound, mode of healing of . 279
Yellow fever, effects of civilizationon 252

END OF VOL. XVIII.

C. and J. Adlard, Printers, Bartholomew Close.

